# Comparison of articulating and static spacers regarding infection with resistant organisms in total knee arthroplasty

**DOI:** 10.3109/17453674.2011.581266

**Published:** 2011-09-02

**Authors:** En-Rung Chiang, Yu-Ping Su, Tain-Hsiung Chen, Fang-Yao Chiu, Wei-Ming Chen

**Affiliations:** ^1^Department of Orthopaedics and Traumatology, Veterans General Hospital; ^2^Department of Surgery, School of Medicine, National Yang-Ming University, Taipei, Taiwan; Correspondence Y-PS: ericypsu@gmail.com

## Abstract

**Introduction:**

The result of treatment of infections involving antibiotic-resistant organisms in total knee arthroplasty (TKA) is often poor. We evaluated the efficacy of 2-stage revision in TKAs infected with resistant organisms and compared the clinical outcomes with articulating and conventional static spacers, in terms of both infection control and function.

**Methods:**

In a prospective manner, from June 2003 to January 2007 selected patients with a TKA infected with resistant organisms were enrolled and treated with 2-stage re-implantation. The 45 patients were divided into 2 groups: group A (23 patients) implanted with the articulating spacers and group S (22 patients) implanted with static spacers. All patients followed the same antibiotic protocols and had the same re-implantation criteria. The efficacy of infection control was evaluated using re-implantation rate, recurrence rate, and overall success rate. The functional and radiographic results were interpreted with the Hospital of Special Surgery (HSS) knee score and the Insall-Salvati ratio.

**Results:**

With mean 40 (24–61) months of follow-up, 22 of 23 knees were re-implanted in group A and 21 of 22 were re-implanted in group S. Of these re-implanted prostheses, 1 re-infection occurred in group A and 2 occurred in group S. Range of motion after re-implantation, the final functional scores, and the satisfaction rate were better in group A. One third of the patients in group S, and none in group A, had a patella baja.

**Interpretation:**

After 2-stage re-implantation of TKAs originally infected with resistant organisms, the clinical outcome was satisfactory—and similar to that reported after treatment of TKAs infected with low-virulence strains. Treatment with an articulating spacer resulted in better functional outcome and lower incidence of patella baja.

Two-stage re-implantation has been reported to give better infection control than 1-stage revision surgery (Goldman et al. 1996) and it is widely used for chronic total knee arthroplasty (TKA) infection. During the interim period between resection arthroplasty and re-implantation, antibiotic-laden bone cement spacer is used. The static spacers are usually fashioned as a block and keep the knee in extension, which may lead to contraction of the extensor mechanism and joint capsule, making the subsequent exposure for re-implantation difficult. The range of motion and functional outcome after 2-stage revision is poorer than that after aseptic revision of TKA (Jacobs et al. 1989, Wang et al. 2004). With a smooth and congruent interface, articulating spacers are designed to allow motion to prevent soft tissue contracture and improve the daily activity of patients in the hope of better final function.

Many studies have compared the infection control and final function between treatment with articulating spacers and static spacers (Marsi et al. 1997, Fehring et al. 2000, Emerson et al. 2002, Jacobs et al. 2009, Jämsen et al, 2009). However, patients in most of these studies were not stratified according to infecting organisms and antibiotic treatment. The results of clinical studies comparing infections with antibiotic-resistant organisms and low-virulence organisms that were treated with 2-stage surgery have varied widely (Hirakawa et al. 1998, Kilgus et al. 2002, Volin et al. 2004, Mittal et al. 2007). The increasing prevalence of resistant organisms makes it important to evaluate the clinical efficacy of current treatment methods in patients who are infected with these organisms.

We have previously reported an easy technique for making articulating spacers during surgery, with low cost and good quality (Su et al. 2009). In that study, we noted satisfactory results for infections with antibiotic-resistant organisms. In this prospective controlled study, we evaluated the efficacy of 2-stage revision for TKA infected with resistant organisms—methicillin-resistant *Staphylococcus aureus* (MRSA) and methicillin-resistant coagulase-negative Staphylococcus (MRCNS)—and compared the clinical outcomes from using articulating spacers or conventional static spacers together with a consistent antibiotics policy.

## Methods

**Figure 1.**
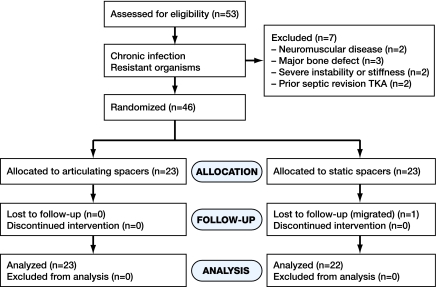
Flow diagram of enrollment.

We included patients who had type-II (late chronic) total knee infection with documented antibiotic-resistant Staphylococcus strains: MRSA or MRCNS. The patients met at least one of the following criteria of deep infection: infective loosening, drainage sinus, or recurrent infection after debridement. Patients who suffered from neuromuscular disease precluding active motion, who had had previously failed septic revision TKA, who had major bone defects, who had complete tear of collateral ligament, or who had knee motion of less than 45 degrees were excluded. From June 2004 to January 2007, in a prospective manner, 46 selected patients were split into 2 groups by selecting every other patient. The process of designation was open to the medical team only. In group A, 23 patients were implanted with in-house articulating spacers and in group S, 23 patients had block-type static spacers. 1 patient in group S moved away before re-implantation and was lost to follow-up (Figure 1 and Table 1). 6 patients in group A were also reported in the pilot study describing the technique of making articulating spacers used in this study (Su et al. 2009).

**Table 1.** Demographic data

	Articulating spacer	Static spacer
	(n = 23)	(n = 22)
Age (years)	71 (65–78)	72 (67–80)
Sex (female / male)	13 / 10	10 / 12
Infected TKA	19	18
Infected revised TKA	4	4
After debridement	13	9
Medical co-morbidities	9	11
Diabetes mellitus	6	7
PAOD	1	1
End-stage renal disease	2	1
Rheumatoid arthritis	1	2
Others	1 breast cancer	1 liver cirrhosis, Child A
Bacteriology		
MRSA	19	20
MRCNS	4	2	
Intravenous antibiotics (weeks)	3.4 (2.5–4.5)	3.1 (2.5–4.5)
Oral antibiotics (weeks)	8.3 (4–11)	8.1 (4–10)
Spacer stage (months)	3.4 (2.5–5)	3.1 (2–4)
Re-implantation	22	21
Surgical approach		
Medial parapatellar	22	14
Quadriceps snip	0	6
V-Y plasty	0	1
Mean follow-up (months)	41 (24–61)	40 (24–59)

PAOD: peripheral arterial occlusion disease; MRSA: methicillin-resistant *Staphylococcus aureus*; MRCNS: methicillin-resistant coagulase-negative staphylococci.

During the resection surgery, all the old components, cement, and infective tissue were removed. The sclerotic or cystic bony surface was refreshed with burrs. Then the wound and medullar canal were irrigated thoroughly with pulse lavage. Simplex-P cement (Howmedica, Rutherford, NJ) was used to make spacers.

**Figure 2.**
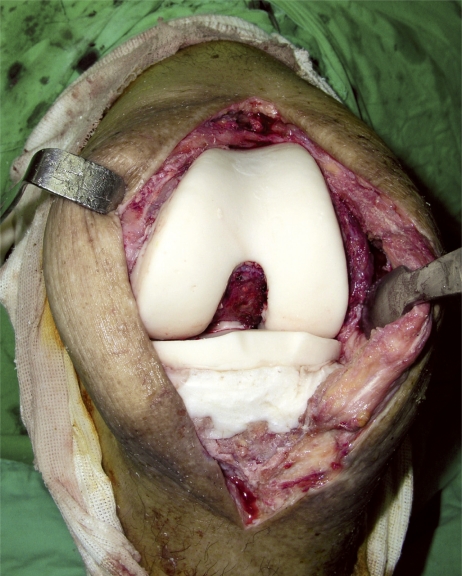
Spacers cast with PDMS molds provide a smooth interface. Care should be taken to balance the gap tension and restore the proper joint line when implanting articulating spacers.

In the group with articulating spacers, 2 g of vancomycin was hand-mixed with each 40-g package of cement. In total, 6 g of vancomycin in 3 packs of cement was used for each case. The articulating spacers were cast intraoperatively with molds fabricated from silicon rubber, polydimethyl siloxane (Coltoflax; Coltène AG, Altstatten, Switzerland) according to the technique already described (Su et al. 2009). Briefly, the doughy putty matrix of polydimethyl siloxane is thoroughly mixed with the catalyst (alkyl silicate) and applied to the surface of a prosthesis collected before surgery for use as a template. It will set in 5 to 10 min. The negative impression made from this material replicates the smooth surface of the prosthesis in detail. To make each set of knee spacers, separate molds for the femoral and insert parts are required. These molds can be re-used and re-sterilized with peroxide plasma. After prosthesis removal, and meticulous debridement and irrigation, the first pack of cement was poured into the prefabricated molds to cast the femoral and insert parts of the spacers. The other 2 packs of cement were used sequentially to fix the spacers. All spacers were loosely fixed to the bone. The joint line was properly restored and care was taken not to balance the gap tension too tight (Figure 2).

In the group with static spacers, 6 g of vancomycin was mixed with 2 packs of cement. Each 40-g package of cement contained 3 grams of vancomycin. The cement was fashioned into a block, which was fitted into extension gap with the knee straight.

After surgery, on limbs with static spacers, long leg splinting was applied followed by casting until re-implantation. Patients with the articulating spacers had a long leg splint for 1 week and they were then encouraged to perform range-of-motion exercises as tolerated, and to walk with protective weight bearing. Continuous passive motion was used during hospitalization.

All patients followed a similar antibiotics protocol after surgery. Intravenous antibiotics, vancomycin or teicoplanin, were given for at least 2 weeks or until clinical infection control was observed and CRP had decreased to less than 2.0 mg/dL. Oral sodium fusidate tablets were prescribed on an outpatient basis for at least 4 weeks or more, until the CRP value returned to normal. The criterion for re-implantation was CRP of < 1.0 mg/dL two weeks after discontinuing the oral antibiotics, and without any clinical signs of infection.

Re-implantation was done using a medial parapatellar approach. Quadriceps snip or V-Y lengthening was used only when adequate exposure could not be obtained otherwise. The spacers were removed and soft tissue between the cement and bone junction was sent for frozen sectioning (considered positive for infection with ≥ 10 polymorphonuclear leukocytes per high-power field). The revision prostheses used were all of the non-hinged type (LCCK; Zimmer, Warsaw, IN). Vancomycin-impregnated cement was used to fix the prosthesis with 1 g antibiotic per 40-gram package of cement. The management after re-implantation was similar to that for aseptic revision surgery except that there were 6 additional weeks of oral sodium fusidate.

The change in patella tendon length was measured by comparing the Insall-Salvati ratio (Insall and Salvati 1971) between stages. Patella baja was defined as a ratio of less than 0.8. Knee function was evaluated with the Hospital of Special Surgery (HSS) knee score (Insall et al. 1976). Evaluations were performed by an independent investigator.

### Statistics

Continuous variables such as change in Insall-Salvati ratio before and after each type of treatment and the functional scores were analyzed with a Student t-test. The association between type of treatment and each categorical variable (infection control, re-infection, and presence of patella baja) was analyzed with Fisher's exact test. Differences were considered to be statistically significant at p-values of < 0.05.

## Results

The distribution of sex, age, and clinical background was similar between the groups. Group A had 19 cases of MRSA and 4 cases of MRCNS infection while group S had 20 cases of MRSA and 2 cases of MRCNS infection. These resistant strains were all sensitive to vancomycin.

The intravenous vancomycin was administrated throughout the entire hospitalization period after resection surgery, which was about 3 weeks on average for both groups. Oral antibiotics were used for mean 8 weeks in both groups. All the patients tolerated the antibiotics well.

The average interval between resection arthroplasty and re-implantation was 3.4 months in group A and 3.1 months in group S. During the spacer stage, the patients in group A achieved mean 83 degrees of knee flexion (maximum 105 degrees). They could sit comfortably with bent knees and walk with partial weight bearing. Crepitating sound from the knee was felt by all patients, and various degrees of cogwheel motion were also noted, especially in the first few weeks. However, the phenomenon disappeared within a month. Only 1 patient experienced knee lock at flexion during hospitalization, which resolved after skin traction. No gross instability of the knee was noted.

All but 2 patients (1 in each group) were re-implanted. The failed patient in group A had a fluctuating CRP level, and the infection recurred with a discharging wound during the period without drugs. He had another debridement to retrieve the residual cement in the femoral canal, and was implanted with a new articulating spacer. Re-implantation was done successfully after 3 months. The failed patient in group S had a persistent discharging sinus and was treated with arthrodesis.

All of the revision surgeries in group A (22 patients) were carried through medial parapatellar arthrotomy, while 7 of 21 patients in group S required extensor interruption to obtain adequate exposure. The average surgical time was 121 min in group A and 163 min in group S. Frozen sections and bacterial cultures were all negative at re-implantation.

The functional results at mean 40 (24–61) months of follow-up were better in group A in terms of HSS knee score and knee motion (Table 2). There was no substantial extension lag in group A, while those who had extensor interruption procedures in group S had various degrees of extensor lag. The Insall-Salvati ratio in group A was similar before and after 2-stage revision. However, in group S, it decreased from 0.95 to 0.81 after revision and one third of the patients had a patella baja, as compared to none before resection. The satisfaction rate for the mobile spacer was statistically significantly higher in group A. In group S, inconvenience from a straight stiff knee was the commonest complaint followed by problems from the cast, such as pressure sores and dermatitis.

**Table 2.** Clinical results

	Articulating spacer	Static spacer
	(n = 23)	(n = 22)
Re-infection rate	1/22	2/21
Success rate	21/23	19/22
ROM at spacer stage (°)	84 (50–105)	0
Post-revision ROM (°)	113 (95–125)	85 (70–100) **^b^**
Post-revision HSS score	90 (86–94)	82 (81–88) **^b^**
Patella baja		
Preoperative (%)	0	0
Mean ISR **^a^**	0.98	0.95
Postoperative (%)	0	33 **^b^**
Mean ISR **^a^**	0.93	0.81 **^b^**
Satisfaction rate	21/23	7/22 **^b^**

**^a^** Insall-Salvati ratio.

**^b^** p < 0.05

The number of re-infections at the latest follow-up was 1 in group A and 2 in group S. The one patient in group A had had an infected revision TKA before the 2-stage treatment while the 2 patients in group S had an infected primary and revision TKA, respectively. All 3 cases fulfilled the re-implantation criteria and their frozen sections during the index surgery were negative for active infection. Only 1 of them (in the S group) had the same pathogen. The other 2 failures had negative cultures but had obvious clinical presentation (one with a discharging wound, the other with an erythmatous swelling along with an elevated white count in the joint aspiration).

## Discussion

With numerous different protocols and various types of bacteria reported, the success rate of 2-stage total knee revision varies between from 82% and 100% (Rosenberg et al. 1988, Windsor et al. 1990, Goldman et al. 1996, Jacobs et al. 2009, Jämsen et al. 2009). The prognosis for arthroplasties infected with resistant organisms has been reported to be poor. In a retrospective study of infected arthroplasties in 70 patients (Kilgus et al. 2002), the success rates by surgical treatment were only 9/19 for hip replacements and 3/16 for knee replacements infected with MRSA or methicillin-resistant *Staphylococcus epidermidis* (MRSE). Hirakawa et al. (1998) reported a success rate of 12/18 after two-stage revision for total knee arthroplasty infected with resistant organisms, while it was 20/24 for those infected with low-virulence organisms. However, some authors have reported better results. In a comparative study involving 46 patients, Volin et al. (2004) found 1 failure out of 9 after 2-stage re-implantation for total joints infected with methicillin-resistant organisms, as compared to 2 out of 37 for methicillin-sensitive organisms. In a multicenter study involving 37 patients who had a total knee arthroplasty infected with resistant organisms and who underwent 2-stage reimplantation, Mittal et al. (2007) reported eradication of the infection in 33 patients, when those who were re-infected with an organism different from the original one were included. In our study, 40 of the 45 patients became infection-free after re-implantation, which supports the efficacy of 2-stage reimplantation for TKAs infected with resistant organisms.

The potential advantages of articulating spacers are a facilitated re-implantation and better final functional results. However, it is still unclear whether the efficacy of infection control is comparable between articulating and static spacers. In many series using articulating spacers with case numbers exceeding 20, the re-infection rate has ranged between 0% and 12% (Hofmann et al. 1995, 2005, Haddad et al. 2000, Emerson et al. 2002, Durbhakula et al. 2004, Evans 2004, Meek et al. 2004), which is similar to the results in series with static spacers (Jacobs et al. 1989, Windsor et al. 1990, Goldman et al. 1996, Fehring et al. 2000, Emerson et al. 2002, Wang et al. 2004). Fehring et al. (2000) and Emerson et al. (2002) reported similar infection recurrence rates for use of articulating and static spacers in their comparative studies. Jacobs et al (2009) reviewed 18 studies on infected prostheses treated with two-stage re-implantation, and found re-infection rates (after pooling of cases) of 6% (21/361) for mobile spacers and 8% (10/122) for static spacers. However, to our knowledge there have been few prospective studies comparing these two different approaches, solely based on resistant organisms. In our series, the success rates were similar for articulating and static spacers.

Failure to provide an anatomic surface in articulating spacers may cause instability. The impression-taking technique described in this study used polydimethyl siloxane (PDMS) and Coltoflax to make molds. PDMS is a non-toxic, biocompatible material that is commonly used in the process of microfabrication for tissue engineering. It is also widely used in dental procedures with its property of easy handling. The molds have a smooth surface that exactly reflects the shape of the original prosthesis. It does not adhere to the cement, so the use of any lubricant is avoided. With these spacers, we found no gross instability or spacer breakage. All patients could walk with partial weight bearing and sit comfortably with bent knees. Thus, higher satisfaction rate was observed in patients with better joint ROM.

Although antibiotic-laden bone cement has been used for many decades, there is no consensus about the amount of antibiotics that should be mixed into the cement spacers. We mixed 2 g of antibiotics with 40 g of cement, and each knee contained 6 g of antibiotics in total. This dosage is at the lower range of the usual recommended dose (Hanssen and Spangehl 2004, Jiranek et al. 2006). We did, however, achieve a good rate of eradication of infection with this dosage.

Two-stage revision with static spacers has been reported to be frequently associated with complications due to poor bone quality, patella tendon avulsion, extension lag, flexion limitation, and extension instability (Rosenberg et al. 1988, Windsor et al. 1990, Goldman et al. 1996, Meek et al. 2004). In our study, none of the patients with articulating spacers had these complications. Gap tensioning was done during the spacer surgery, so there was no need for extensive scar release during the re-implantation procedure, which resulted in shorter operation time. On the other hand, patients with static spacers required more extensor mechanism interruption, which may result in extension lag; one third of patients with static spacers developed patella baja after a long-term casting. Not surprisingly, the patients treated with articulating spacers achieved much better functional results than those who were treated with static spacers.

In summary, 2-stage re-implantation with articulating spacers impregnated with vancomycin achieved an infection eradication rate similar to that with static spacers for TKAs infected with methicillin-resistant *Staphylococcus aureus* or methicillin-resistant coagulase-negative *Staphylococcus.* The use of articulating spacers avoided the development of patella baja and complications from the extensor mechanism, which led to better functional results.

## References

[CIT0001] Durbhakula SM, Czajka J, Fuchs MD, Uhl RL (2004). Antibiotic-loaded articulating cement spacer in the 2-stage exchange of infected total knee arthroplasty. J Arthroplasty.

[CIT0002] Emerson RH, Muncie M, Tarbox TR, Higgins LL (2002). Comparison of a static with a mobile spacer in total knee infection. Clin Orthop.

[CIT0003] Evans RP (2004). Successful treatment of total hip and knee infection with articulating antibiotic components: A modified treatment method. Clin Orthop.

[CIT0004] Fehring TK, Odum S, Calton TF, Mason JB (2000). Articulating versus static spacers in revision total knee arthroplasty for sepsis. Clin Orthop.

[CIT0005] Goldman RT, Scuderi GR, Insall JN (1996). Two-stage reimplantation for infected total knee replacement. Clin Orthop.

[CIT0006] Haddad FS, Masri BA, Campbell D, McGraw RW, Beauchamp CP, Duncan CP (2000). The PROSTALAC functional spacer in two-stage revision for infected knee replacement. J Bone Joint Surg (Br).

[CIT0007] Hanssen AD, Spangehl MJ (2004). Practical applications of antibiotic-loaded bone cement for treatment of infected joint replacements. Clin Orthop.

[CIT0008] Hirakawa K, Stulberg BN, Wilde AH, Bauer TW, Secic M (1998). Results of 2-stage reimplantation for infected total knee arthroplasty. J Arthroplasty.

[CIT0009] Hofmann AA, Kane KR, Tkach TK, Plaster RL, Camargo MP (1995). Treatment of infected total knee arthroplasty using an articulating spacer. Clin Orthop.

[CIT0010] Hofmann AA, Goldberg T, Tanner AM, Kurtin SM (2005). Treatment of infected total knee arthroplasty using an articularing spacer: two to 12-year experience. Clin Orthop.

[CIT0011] Insall J, Salvati E (1971). Patella position in the normal knee joint. Radiology.

[CIT0012] Insall JN, Ranawat CS, Aglietti P, Shine J (1976). A comparison of four models of total knee-replacement prostheses. J Bone Joint Surg (Am).

[CIT0013] Jacobs MA, Hungerford DS, Krackow KA, Lennox DW (1989). Revision of septic total knee arthroplasty. Clin Orthop.

[CIT0014] Jacobs C, Christensen CP, Berend ME (2009). Static and mobile antibiotic-impregnated cement spacers for the management of prosthetic joint motion. J Am Acad Orthop Surg.

[CIT0015] Jämsen E, Stogiannidis I, Malmivaara A, Pajamäki J, Puolakka T, Konttinen YT (2009). Outcome of prosthesis exchange for infected knee arthroplasty: the effect of treatment approach. A systematic review of the literature. Acta Orthop.

[CIT0016] Jiranek WA, Hanssen AD, Greenwald AS (2006). Antibiotic-loaded bone cement for infection prophylaxis in total joint replacement. J Bone Joint Surg (Am).

[CIT0017] Kilgus DJ, Howel DJ, Strang A (2002). Results of periprosthetic hip and knee infections caused by resistant bacteria. Clin Orthop.

[CIT0018] Marsi BA, Duncan CP, Beauchamp CP, Engh GA, Rorabeck CH (1997). The modified two-stage exchange arthroplasty in the treatment of the infected total knee replacement: the Prostalac system and other articulated spacers. Revision total knee arthroplasty.

[CIT0019] Meek RM, Dunlop D, Garbuz DS, McGraw R, Greidanus NV, Masri BA (2004). Patient satisfaction and functional status after aseptic versus septic revision total knee arthroplasty using the PROSTALAC articulating spacer. J Arthroplasty.

[CIT0020] Mittal Y, Fehring TK, Hanssen A, Marculescu C, Odum SM, Osmon D (2007). Two-stage reimplantation for periprosthetic knee infection involving resistant organisms. J Bone Joint Surg (Am).

[CIT0021] Rosenberg AG, Haas B, Barden R, Marquez D, Landon GC, Galante JO (1988). Salvage of infected total knee arthroplasty. Clin Orthop.

[CIT0022] Su YP, Lee OK, Chen WM, Chen TH (2009). A facile technique to make articulating spacers for infected total knee arthroplasty. J Chin Med Assoc.

[CIT0023] Volin SJ, Hinrichs SH, Garvin KL (2004). Two-stage reimplantation of total joint infections. A comparison of resistant and non-resistant organisms. Clin Orthop.

[CIT0024] Wang CJ, Hsieh MC, Huang TW, Wang JW, Chen HS, Liu CY (2004). Clinical outcome and patient satisfaction in aseptic and septic revision total knee arthroplasty. Knee.

[CIT0025] Windsor RE, Insall JN, Urs WK, Miller DV, Brause BD (1990). Two-stage reimplantation for the salvage of total knee arthroplasty complicated by infection. Further follow-up and refinement of indications. J Bone Joint Surg (Am).

